# An alternate mode of oligomerization for *E. coli* SecA

**DOI:** 10.1038/s41598-017-11648-5

**Published:** 2017-09-18

**Authors:** Aliakbar Khalili Yazdi, Grant C. Vezina, Brian H. Shilton

**Affiliations:** Department of Biochemistry, University of Western Ontario, London, Ontario, N6A 5C1 Canada

## Abstract

SecA is the ATPase of preprotein translocase. SecA is a dimer in solution and changes in its oligomeric state may function in preprotein translocation. The SecA-N68 construct, in which the C-terminal helical domains of SecA are deleted, was used to investigate the mechanism of SecA oligomerization. SecA-N68 is in equilibrium between monomers, dimers, and tetramers. Subunit interactions in the SecA-N68 tetramer are mediated entirely by unstructured regions at its N- and C-termini: when the termini are deleted to yield SecA-N68∆NC, the construct is completely monomeric. This monomeric construct yielded crystals diffracting to 2.6 Å that were used to solve the structure of SecA-N68, including the “preprotein crosslinking domain” (PPXD) that was missing from previous *E. coli* SecA structures. The SecA-N68 structure was combined with small angle X-ray scattering (SAXS) data to construct a model of the SecA-N68 tetramer that is consistent with the essential roles of the extreme N- and C-termini in oligomerization. This mode of oligomerization, which depends on binding of the extreme N-terminus to the DEAD motor domains, NBD1 and NBD2, was used to model a novel parallel and flexible SecA solution dimer that agrees well with SAXS data.

## Introduction

The bacterial General Secretory System is centred around two essential components: the SecYEG complex, which forms a pore through the cytoplasmic membrane, and SecA, an ATPase that couples ATP binding and hydrolysis to translocation of an unfolded preprotein substrate through the SecYEG pore^1–3^. The mechanism by which SecA catalyzes preprotein movement is not known; in this regard, a critical question for any potential mechanism is whether the translocating system incorporates a single SecA subunit, or whether multiple SecA subunits are involved^4^. Crystal structures of the SecA-SecYEG complex have illuminated the interaction between SecA and SecYEG at atomic resolution^5,6^. From these structures and biochemical studies a translocation mechanism that involves only a single SecA-SecYEG complex can be envisioned^7–11^. In such a mechanism, ATP binding and hydrolysis by SecYEG-bound SecA drives conformational changes in the complex that mediate preprotein movement through SecYEG.

Alternative mechanisms incorporate multiple SecA molecules in the translocation reaction^12–18^, although the roles of the additional SecA molecules remain speculative. Evidence for the involvement of multiple SecA subunits also comes from the accessory translocase system in *M. tuberculosis*, which harbours two SecA genes, *secA1* and *secA2*. SecA1 is essential and most closely resembles the single, essential SecA found in *E. coli*, whereas SecA2 has a slightly altered domain structure and is essential for the secretion of only a few particular preproteins^19^. SecA2-mediated secretion also requires SecA1, and so for secretion of SecA2-dependent preproteins, at least two SecA molecules are involved^20^. In the case of the general secretory system of *E. coli*, the available evidence does not provide a conclusive answer as to the number of SecA subunits required for translocation. Attempts to address this question have been confounded by the fact that SecA exists as a dimer in solution^21–24^, but given the changes in SecA oligomerization required for its interaction with SecYEG, it is not clear whether the solution dimer is related to oligomeric species that may participate in the translocation reaction^13,25^. It is conceivable that interactions between SecA protomers could function to regulate ATPase activity as well as binding between SecA and the preprotein during translocation.

Potential functions for the SecA dimer lead naturally to questions about its structure and the nature of the dimer interface. In this regard, the available crystal structures have not provided a consistent answer because the potential dimers found in SecA crystals are all different. Studies of SecA in solution, by cross-linking^26^, hydrogen-deuterium exchange^27^, and FRET^22,23^ are not wholly consistent with any of the available crystal structures, although the data are most consistent with the dimer in the original *B. subtilis* SecA structure^28^. In this antiparallel dimer, the N-terminal and C-terminal domains interact, and the extreme N-terminus interacts with the C-terminal domains to form part of the dimer interface. Therefore, this dimer structure is also consistent with observations that the solution dimer is weakened by truncation of the N-terminus^10,14,26,29^. Truncation of the N-terminus also leads to translocation defects, indicating that the N-terminus has a functional role^14,29–32^. The N-terminus is important for interaction of SecA with membranes^30–32^ but an important functional role for the oligomerization of SecA has not been excluded. On this basis, the mechanism by which the N-terminus mediates SecA oligomerization is of interest.

Truncation of *E. coli* SecA at residue 610 produces a construct, SecA-N68, that is monomeric at low concentrations but at higher concentrations forms tetramers^33^. Given the question regarding the number of SecA molecules involved in translocation, the formation of this tetramer was an interesting observation, but the significance of the SecA-N68 tetramer to the translocation reaction has never been clear. In the current work, we have discovered that the formation of the SecA-N68 tetramer is completely dependent on unstructured polypeptide segments at its N- and C-termini. Thus, the N-terminus of SecA is able to mediate oligomer formation in the absence of the C-terminal helical domains. Furthermore, since SecA catalyzes movement of unstructured polypeptide through SecYEG, binding of unstructured polypeptide by SecA-N68 to form a tetramer has potential implications for the translocation reaction. Thus, the SecA-N68 tetramer unites the oligomerization of SecA with binding of unstructured polypeptide, which may be a key part of the translocation reaction. In addition, removal of the termini allowed us to obtain crystals of SecA-N68 that diffracted to 2.6 Å resolution and yielded a structure that includes both the DEAD Motor domains and the preprotein crosslinking domain (PPXD), which was disordered in the original *E. coli* SecA structure^34^. With the SecA-N68 structure and knowledge of the critical roles of the unstructured termini in stabilizing the tetramer, a SAXS-based model of the SecA-N68 tetramer was produced in which the N-terminus interacts with the DEAD motor domains.

By combining our *E. coli* SecA-N68 structure with the available crystal structures, models of the complete *E. coli* SecA protomer were produced based on the available conformations of SecA in the Protein Data Bank. Solution structures for the SecA-N68 tetramer and the SecA-N95 dimer were evaluated based on small-angle X-ray scattering (SAXS) data. Modelling of SecA-N95 against the SAXS data suggests that the dimer seen in the original *B. subtilis* structure^28^ may not be representative of the *E. coli* solution dimer. An alternate SecA solution dimer is suggested that is in better agreement with the SAXS data, and in which the N-terminus interacts with the DEAD motor domains. Therefore these structural studies point towards a novel interaction between the N-terminus and the DEAD motor domains that could play a role in SecA oligomerization.

## Results

### Crystal Structure of SecA-N68

**Figure 1:**
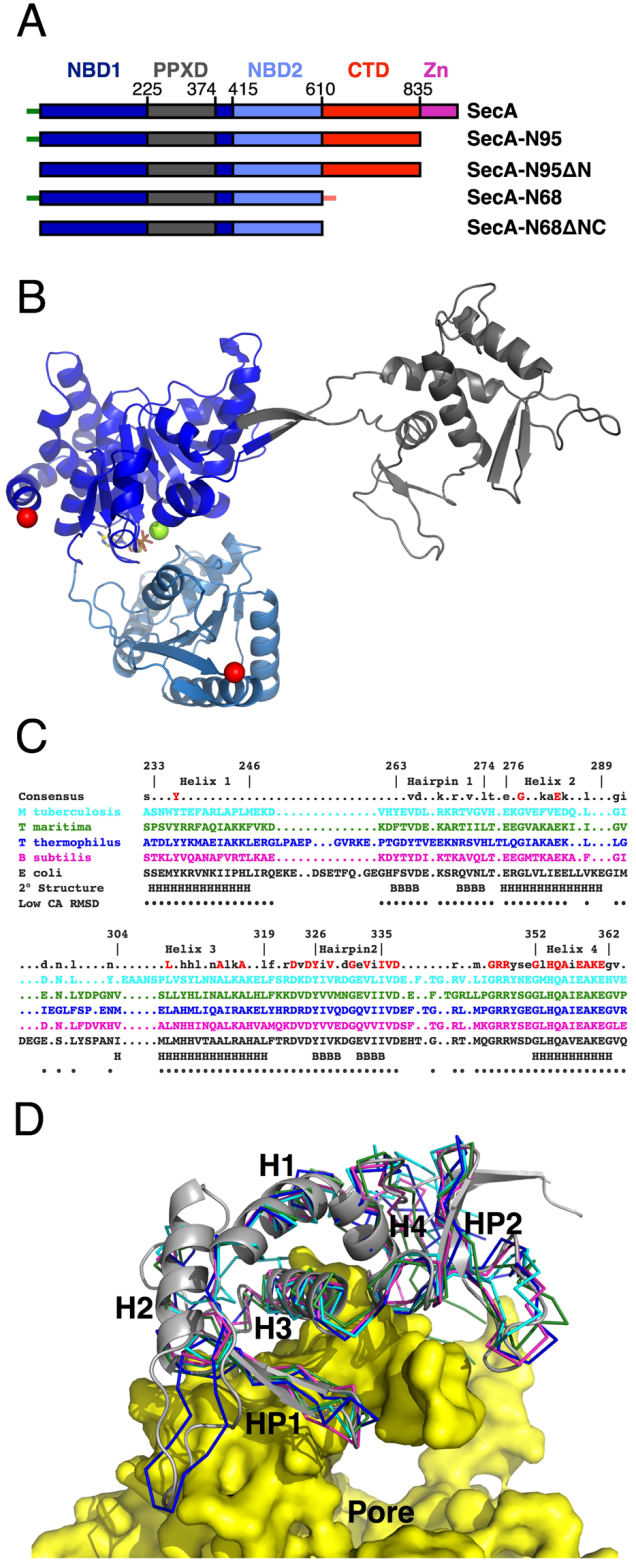
Structure of SecA-N68 (**A**) Domain structure of SecA and deletion constructs. Full length SecA consists of nucleotide binding domain 1 (NBD1); the preprotein cross-linking domain (PPXD), which is connected to NBD1 through a β-hairpin linker; NBD2; the C-terminal domains (CTD), which can be further sub-divided into the helical scaffold domain (HSD), residues 611 to 670 and 755 to 835 and helical wing domain (HWD), residues 671 to 754; finally there is the zinc binding domain, which consists of a 22 residue zinc-binding motif at the extreme C-terminus, connected to residue 835 by an unstructured linker. The unstructured N-terminus of SecA is illustrated by the short green line, and has the sequence MLIKLLTKVFGSRN; in some constructs, the N-terminal sequence included a hexahistidine tag with sequence MHHHHHHLTKVFGSRN. An unstructured C-terminal sequence (short orange line) was also present in SecA-N68, which, starting from residue 597, had the sequence RIFASDRVSGMMRK. (**B**) Structure of SecA-N68∆NC, with domains colour-coded as in Panel A. The structure also contained Mg^2+^ (green sphere) and ADP (sticks), bound between NBD1 and NBD2. (**C**) Structure-based sequence alignment of the PPXDs from the SecA molecules of 5 different organisms: *M. tuberculosis* (1NKT)^63^, *T. maritima* (3JUX)^48^, *T. thermophilus* (2IPC)^64^, *B*. *subtilis* (1TF5)^11^, and *E. coli*. The numbering and secondary structure (H for helix, B for strand) is for *E. coli* SecA. Residues that have a relatively small CA RMSD between the 5 structures are indicated with black dots. (**D**) Superposition of PPXD structures. The *E. coli* PPXD from the SecA-N68∆NC structure is shown as a grey ribbon and the other four structures (as in Panel C) are shown as CA-traces. The 5 superposed PPXD structures have been mapped onto the SecYEG-bound PPXD in the *T. maritima* SecA-SecYEG complex (3DIN)^6^.

The crystal structure of *E. coli* SecA has been solved^34^, but the “Preprotein Cross-Linking Domain” (PPXD) was disordered and not built as part of the structure. To obtain a structure of the *E. coli* PPXD, we used a deletion construct, “SecA-N68” that lacks the C-terminal domains, including the Helical Wing Domain (HWD), the Helical Scaffold Domain (HSD), and a linker connected to the Zinc Binding Domain (ZBD; Fig. 1A). SecA-N68 cannot catalyze translocation, but it retains functionality in that it binds with high affinity to SecYEG in membranes^35^, has a constitutive ATPase activity, and binds peptides corresponding to the signal sequences of LamB and OmpA^36^. To obtain crystals of SecA-N68, we removed unstructured regions at the N- and C-termini so that the construct included residues 15 to 590, which we term “SecA-N68∆NC”. In addition, entropy-reducing mutations to alanine^37^ were introduced in two areas, one containing E55, K56, and E58, and a second at E196 and E197. Crystals of the SecA-N68∆NC construct diffracted to 2.6 Å resolution; the structure was solved using molecular replacement and refined to an *R*_free_ of 0.25 (Table 1).

**Table 1:** Crystallographic Data and Refinement Statistics for SecA-N68∆NC

Parameter	^c^SecA-N68∆NC
Wavelength (Å)	1.10554
Space Group	P2_1_
Unit Cell Dimensions (Å)	a = 67.06, b = 64.45, c = 87.97 β = 105.85°
Resolution (Å)	17.92 - 2.60
^a^ *R*_sym_	0.120 (0.511)
^a^ *I*/σ*I*	8.8 (2.6)
^a^Completeness	99.4 (96.8)
Multiplicity	4.0
Unique Reflections	22456
*R*_work_/*R*_free_	0.1956/0.2497
Ramachandran Plot (%)	
Most Favoured	90.5
Additionally Allowed	9.5
Generously Allowed	0
Disallowed	0
RMS Deviations	
Bond Lengths (Å)	0.006
Bond Angles (deg)	0.769
Dihedral Angles (deg)	14.028
Mean ADP Values (Å^2^)	
Protein	48.2
Solvent	29.2
Mg^2+^-ADP	24.6

^a^Values in parentheses refer to highest resolution shell.

^b^Ramachandran plot statistics were calculated using PROCHECK^67^.

^c^Deposited in the PDB with code 5K9T.

The structure of SecA-N68∆NC bound to Mg^2+^-ADP (Fig. 1B) contains the DEAD Motor domains, NBD1 and NBD2, in the same conformation observed for other SecA structures, with the exception of the *E. coli* SecA structure which was crystallized in the absence of bound nucleotide and in which NBD1 and NBD2 adopt a more open conformation^34^. The β-hairpin anchoring the PPXD to NBD1 is in a fully extended conformation that has not been observed in previous crystal structures, where the hairpin is typically bent to facilitate interaction of the PPXD with the C-terminal helical domains. The *E. coli* PPXD brings to five the number of structurally characterized PPXDs. A structure-based sequence alignment of the five PPXDs from different organisms is shown in Fig. 1C and the corresponding superposition in Fig. 1D where the PPXDs are mapped onto the structure of *T. maritima* SecYEG^6^. The core PPXD structures are conserved throughout, with some differences in loop regions. The region with the highest level of sequence identity runs from Helix 3 to the C-terminus of the PPXD. These regions interact directly with a large loop from SecYEG^6^. Helix 4 is particularly well-conserved, likely because it plays a central role, interacting directly with SecYEG as well as Helix 1, 3, and Hairpin 2.

### Oligomerization of SecA-N68 is mediated by unstructured termini

Previous work in our laboratory^33^ indicated that an N-terminal histidine-tagged version of SecA-N68 (H_6_-SecA-N68) participated in a monomer-tetramer equilibrium with a *K*_D_ of 63 µM^3^. The biological relevance of this interaction has always been an open question because the nature of the interactions between the SecA-N68 protomers was not obvious. In particular, given the proposed anti-parallel SecA dimer structure observed in *B. subtilis* SecA^28^ it seemed that removal of the C-terminal domains should have produced a completely monomeric construct. The formation of a SecA-N68 tetramer was therefore surprising and difficult to understand. Nevertheless, knowledge of how SecA-N68 self-associates could provide insight into potential interactions between SecA molecules during translocation, and interactions that stabilize the SecA solution dimer.

**Figure 2:**
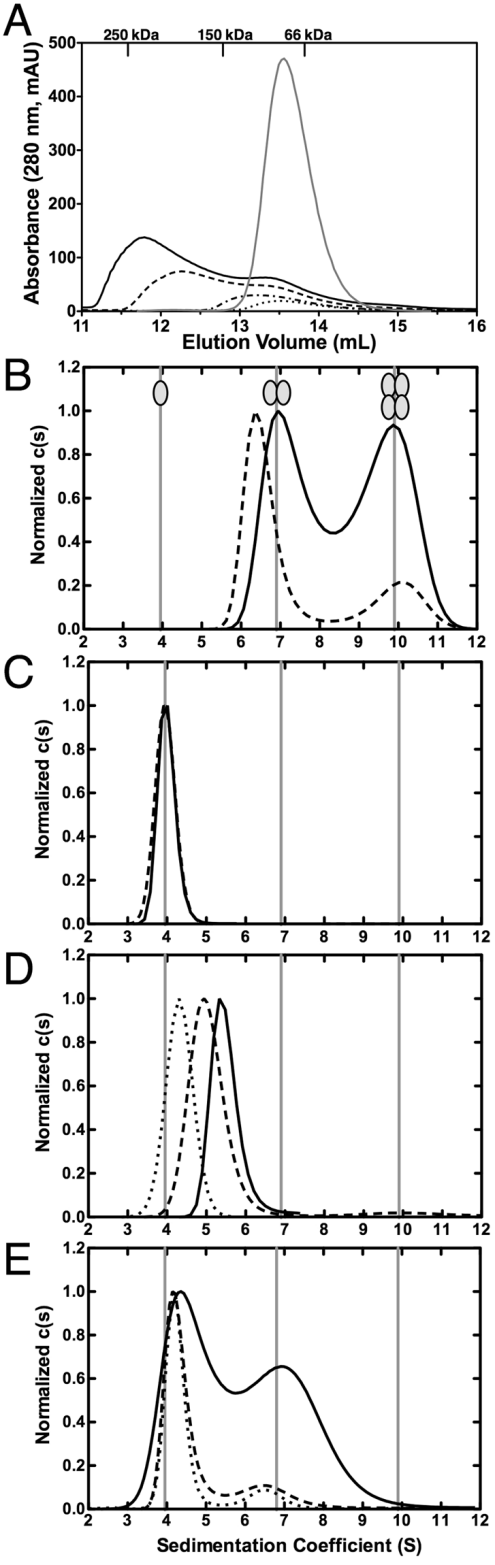
Oligomerization of SecA-N68 is Mediated by Unstructured Termini (**A**) The oligomerization of H_6_-SecA-N68 and SecA-N68∆NC was characterized by gel filtration chromatography. H_6_-SecA-N68 is a SecA construct with an unstructured extreme N-terminal sequence MHHHHHHLTKVFGSRNDRTL (the wild-type sequence is MLIKLLTKVFGSRNDRTL) and also an unstructured C-terminal sequence; SecA-N68∆NC is the same construct, except that the unstructured N- and C-terminal sequences have been removed. H_6_-SecA-N68 was applied to the column at concentrations of 4 mg•mL^−1^ (solid black curve), 2 mg•mL^−1^ (dashed curve), 0.6 mg•mL^−1^ (dashed-dotted curve), and 0.35 mg•mL^−1^ (dotted curve); SecA-N68∆NC (grey curve) was applied at 10 mg•mL^−1^. The elution volume for molecular weight standards is shown on the upper axis. Panels B to E are SedFit analyses from sedimentation velocity AU of 4 different SecA-N68 constructs at a range of concentrations; all constructs were analyzed under identical buffer conditions consisting of 50 mM Tris-HCl pH 7.5, 100 mM KCl, 2 mM EDTA, 5 mM MgCl_2_ and 5 mM TCEP-HCl. (**B**) SecA-N68, with a wild-type N-terminal sequence, was analyzed at concentrations of 1.0 (dashed) and 5.0 (solid) mg•mL^−1^. (**C**) SecA-N68ΔNC, which lacks both unstructured termini, was analyzed at concentrations of 0.1 (dashed) and 4 (solid) mg•mL^−1^. (**D**) Analysis of SecA-N68ΔC, which carries only the wild-type unstructured N-terminus, at concentrations of 0.25 (dotted), 1 (dashed), and 2 (solid) mg•mL^−1^. (**E**) Analysis of SecA-N68ΔN, which carries only the unstructured C-terminus, at concentrations of 0.4 (dotted), 0.6 (dashed), and 4 (solid) mg•mL^−1^.

We could never obtain diffracting crystals of the original SecA-N68 construct, but did manage to produce high quality crystals of SecA-N68∆NC. In the process of doing this, we were surprised to discover that the unstructured termini were solely responsible for mediating oligomerization of H_6_-SecA-N68. That is, SecA-N68∆NC, in which both N- and C-termini had been truncated to remove unstructured polypeptide but is otherwise identical to SecA-N68, is completely monomeric when analyzed by gel filtration chromatography (Fig. 2A). To demonstrate the extent of the difference, H_6_-SecA-N68 migrated with an apparent molecular weight ranging from 89 to 250 kDa at concentrations from 0.04 mg/mL to 4 mg/mL, while SecA-N68ΔNC showed a symmetrical elution peak at a molecular weight of approximately 70 kDa when applied to the column at a concentration of 10 mg/mL.

The role of the unstructured N- and C-termini in the oligomerization of SecA-N68 was investigated in greater detail by analytical ultracentrifugation (AU). Sedimentation velocity experiments using SecA-N68 with a wild-type N-terminus showed that it participates in a dimer-tetramer equilibrium at concentrations of 1.0 and 5.0 mg/mL (Fig. 2B). This behaviour is different from that observed for the histidine-tagged version of SecA-N68 that also contained monomeric species in solution at these concentrations^33^. This shows that the changes in the N-terminal residues due to introduction of the affinity tag weakened the interaction between the protomers. Therefore, it appears that the SecA extreme N-terminus is specifically bound by the NBD1, NBD2, and/or the PPXD, the three domains comprising SecA-N68. As expected from its behaviour when analyzed by gel filtration chromatography, SecA-N68∆NC sedimented as a monomer, showing absolutely no tendency to form oligomers at concentrations up to 4.0 mg/mL (Fig. 2C), the highest concentration tested. These observations demonstrate that the unstructured N- and/or C-terminal peptides mediate oligomerization of SecA-N68.

The previous study of H_6_-SecA-N68 from our laboratory included a low resolution structure for the H_6_-SecA-N68 tetramer, generated from SAXS data^33^. The structure for the tetramer was consistent with D2 symmetry, corresponding to a dimer of dimers. On this basis, and given that SecA-N68∆NC is a monomer, we explored the individual contributions of the N- and C-terminal unstructured sequences to oligomerization of SecA-N68 by deleting either the N- or C-terminus to make SecA-N68∆N and SecA-N68∆C, respectively. Sedimentation velocity analysis of these constructs indicated that SecA-N68∆C equilibrates between a monomer and dimer, with an average molecular weight that increases with concentration (Fig. 2D). SecA-N68∆N is present as discrete monomeric and dimeric species, with the relative amount of the dimer increasing to roughly 50% at 4 mg/mL (Fig. 2E). Thus, both SecA-N68∆N and SecA-N68∆C form a mixture of monomers and dimers in solution.

It is noteworthy that the two constructs, SecA-N68∆N and SecA-N68∆C do not form tetramers, and behave differently from each other when analyzed by sedimentation velocity. In particular, SecA-N68∆N sediments as two discrete peaks corresponding to a monomer and dimer, which is consistent with relatively slow exchange between the two forms^38^. On the other hand, SecA-N68∆C shows a single sharp peak with a molecular weight between that of a monomer and dimer, indicating fast exchange. Therefore, there are two polypeptide binding sites on the SecA-N68 construct, and each one mediates dimerization of SecA-N68 by interacting with either the unstructured N- or C-terminus. Formation of the tetramer requires the presence of both unstructured termini such that two dimers can interact through the two polypeptide binding sites on each protomer.

Small-angle X-ray scattering (SAXS) data provided an *ab initio* model for the SecA-N68 tetramer^33^. The SecA-N68∆NC crystal structure allows use of the SAXS data to search for a tetramer model with the program GLOBSYMM^39^. For the search, D2 (dimer of dimers) symmetry was assumed based on the roles of the N- and C-termini in each mediating a dimer interaction; D2 symmetry is also consistent with the previous *ab initio* SAXS model^33^. GLOBSYMM works by translating and rotating the SecA-N68∆NC structure and evaluating solutions based on their agreement with the SAXS data and the absence of steric clashes. A number of different SecA-N68 tetramer models showed good agreement with the SAXS data; however most of the models were incompatible with the biochemical data because either one or both of the N- and C-termini were directed towards the bulk solvent and not capable of mediating dimer formation.

**Figure 3:**
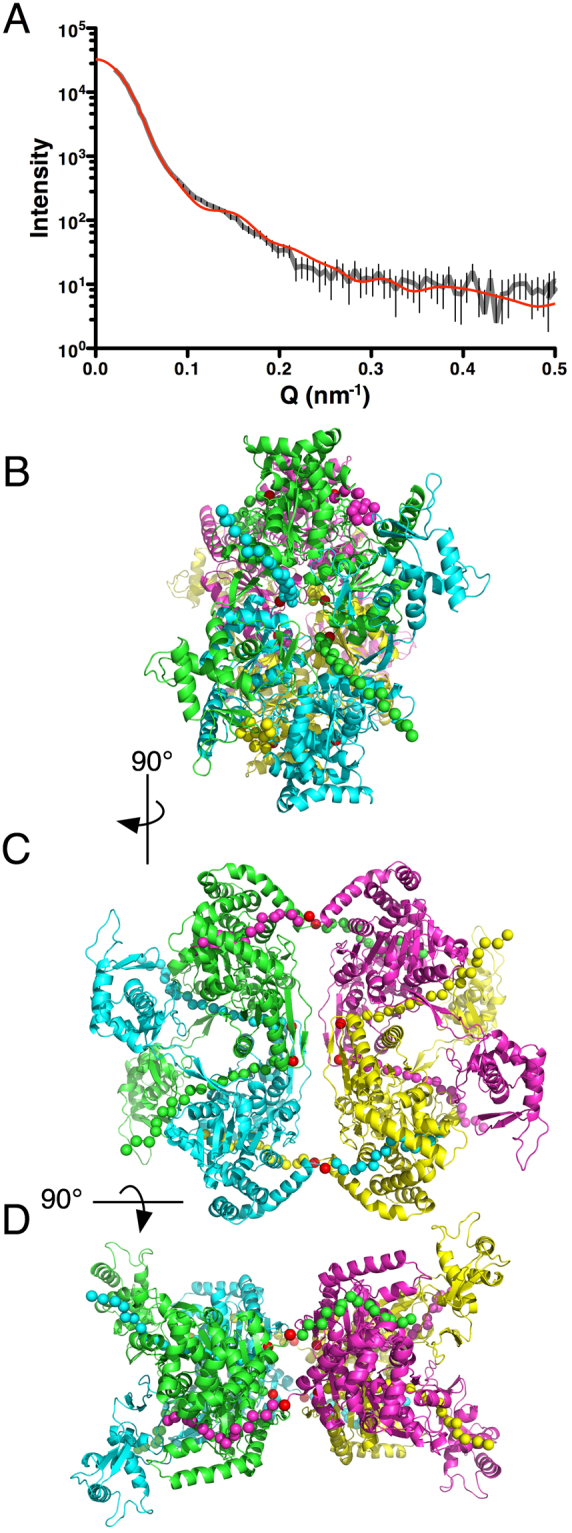
A SAXS-Based Model for the SecA-N68 Tetramer (**A**) The SecA-N68∆NC crystal structure was used with the program GLOBSYMM^39^ to find a tetramer with D2 symmetry that matches the solution SAXS data, which were recorded with SecA-N68 at 6.7 mg/mL^33^. The experimental SAXS data are indicated by the grey curve, with the vertical bars showing the standard deviation of the replicate measurements. The red curve is the scattering from the SecA-N68 tetramer model (Panels B, C, and D), calculated using Crysol^65^. The experimental radius of gyration was 48.2 Å, while the value for the hydrated tetramer model was 48.9 Å; the overall χ^2^ value for the fit of the model to the data was 2.24. The tetramer model is shown from three perspectives related by 90° rotations about a vertical axis (**B** to **C**) and horizontal axis (**C** to **D**). The N- and C-termini of SecA-N68∆NC are shown with red spheres. The additional unstructured residues at the N- and C-termini that mediate tetramer formation in SecA-N68 have been built into the structure to illustrate a potential mode of interaction. The extreme N-terminus is binding in the cleft between NBD1 and NBD2 to mediate dimer formation between the green and magenta protomers, and the yellow and cyan protomers. The extreme C-terminus is binding in a groove next to the hairpin connecting NBD1 to the PPXD, and mediates dimer formation between the green and cyan protomers, and yellow and magenta protomers. Together, the extreme N- and C-termini, illustrated with spheres at CA positions, mediate a dimer-of-dimers tetramer.

**Figure 4:**
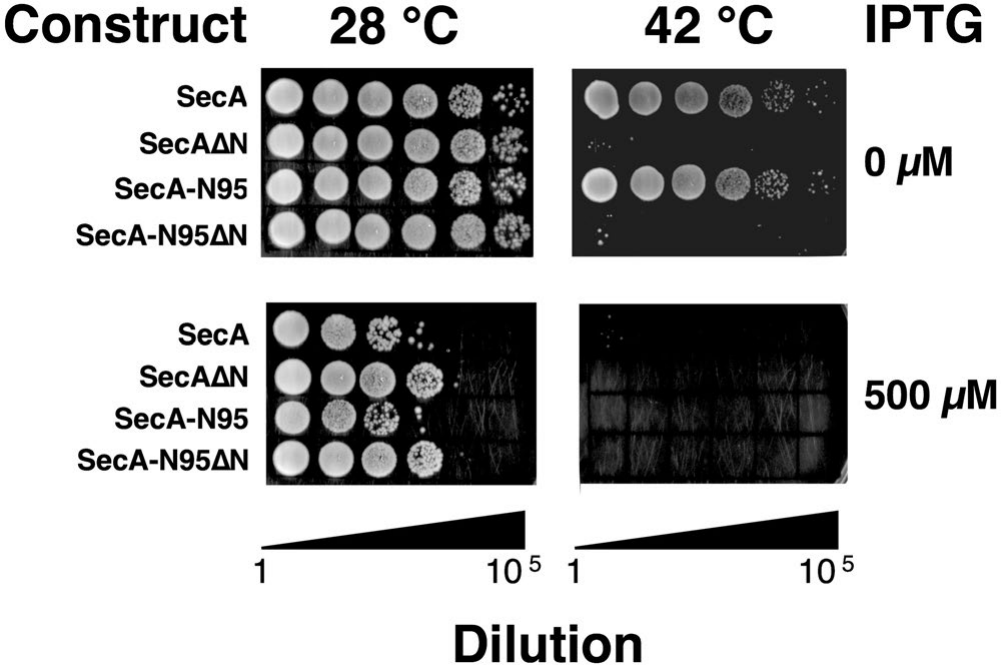
The SecA N-terminus is Essential for Function To test the functionality of SecA constructs, BL21.19(DE3) cells, in which expression of SecA is temperature-sensitive such that the cells do not grow at 42 °C^43^, were transformed with plasmids that express SecA, SecA-N95, or the same constructs but with 14 residues deleted at the N-terminus, SecA∆N and SecA-N95∆N. The cells were grown in liquid culture for 12 hours and diluted to a common OD_600_ of 1, followed by 5 subsequent 10-fold dilutions; 5 µL of the resulting liquid cultures were spotted onto LB-agar and grown at either 28 °C or 42 °C for 30 hours in the absence or presence of IPTG (500 µM).

One tetramer found using GLOBSYMM with the unaltered SecA-N68∆NC structure had both termini oriented so that they could each mediate formation of a dimer, to produce the dimer-of-dimers tetramer. The fit of the tetramer to the SAXS data was excellent, with a χ^2^ value of 2.24 and no steric clashes (Fig. 3). To complete the model, the unstructured N- and C-termini were built into it based on potential peptide binding sites in the SecA DEAD motor domains as assessed using the CABSDock server (http://biocomp.chem.uw.edu.pl/CABSdock/)^40,41^. The docking protocol involves a fragment-based screening approach to locate the most likely peptide binding sites on a protein surface, followed by docking of a peptide sequence to the site. The region most commonly identified as a potential peptide binding site was the cleft between NBD1 and NBD2; another potential site was located in a groove on the opposite side of the NBD1/NBD2 cleft, alongside the β-hairpin that connects NBD1 with the PPXD (for the complete CABSdock results, see Supplemental Tables and Figs 1 to 4). In the tetramer model there are two symmetrical dimers. One of the dimers can be formed by the N-termini binding in the NBD1/NBD2 cleft region. The second dimer is formed by binding of the C-termini to the groove next to the β-hairpin. This mode of binding is illustrated in Fig. 3, panels B to D. This tetramer model is fully compatible with both the solution SAXS data as well as the requirement for a dimer-of-dimers structure that is mediated solely by interactions with the unstructured N- and C-termini; in addition it incorporates the most likely peptide binding sites identified by CABSdock.

### Role of the extreme N-terminus in translocase function and dimerization of SecA-N95

The N-terminus of full-length SecA or SecA-N95, both of which are functional for translocation, has been reported as important both for dimer formation and translocase function^14,29,31^ although another report in which 9 residues at the N-terminus were deleted concluded that the deletion had no effect on dimerization or function^42^. We investigated functional consequences of a 14-residue deletion at the N-terminus by testing whether SecA∆N or SecA-N95∆N could complement a temperature-sensitive SecA strain, BL21-19^43^. Vectors encoding full-length SecA or SecA-N95 were able to complement the function of the tsSecA at the non-permissive temperature of 42 °C; however, the same vectors coding for either SecA∆N or SecA-N95ΔN did not complement SecA function at 42 °C (Fig. 4). Expression of the SecA constructs in both vectors is under the control of the same upstream T7 promotor; in the absence of IPTG, there is sufficient expression of SecA and SecA-N95 to complement the lack of chromosomally-encoded SecA. The same experiment was repeated in the presence of 500 µM IPTG to test whether higher levels of the N-terminal deletion constructs could complement the tsSecA. In fact, higher levels of SecA expression not only failed to facilitate complementation by the N-terminal deletion constructs, but actually abrogated the ability of SecA and and SecA-N95 to complement tsSecA function at 42°, and also decreased viability at 28°. The toxic effects of SecA overexpression have been noted previously and attributed to a requirement for SecA to become a monomer during the translocation cycle^44^. Irrespective of the exact critical function of the N-terminus, whether in membrane binding or SecA oligomerization, the inability SecA∆N and SecA-N95∆N to complement SecA function in BL21–19 cells is broadly consistent with previous work, and demonstrates a critical role for the N-terminal sequence.

**Figure 5:**
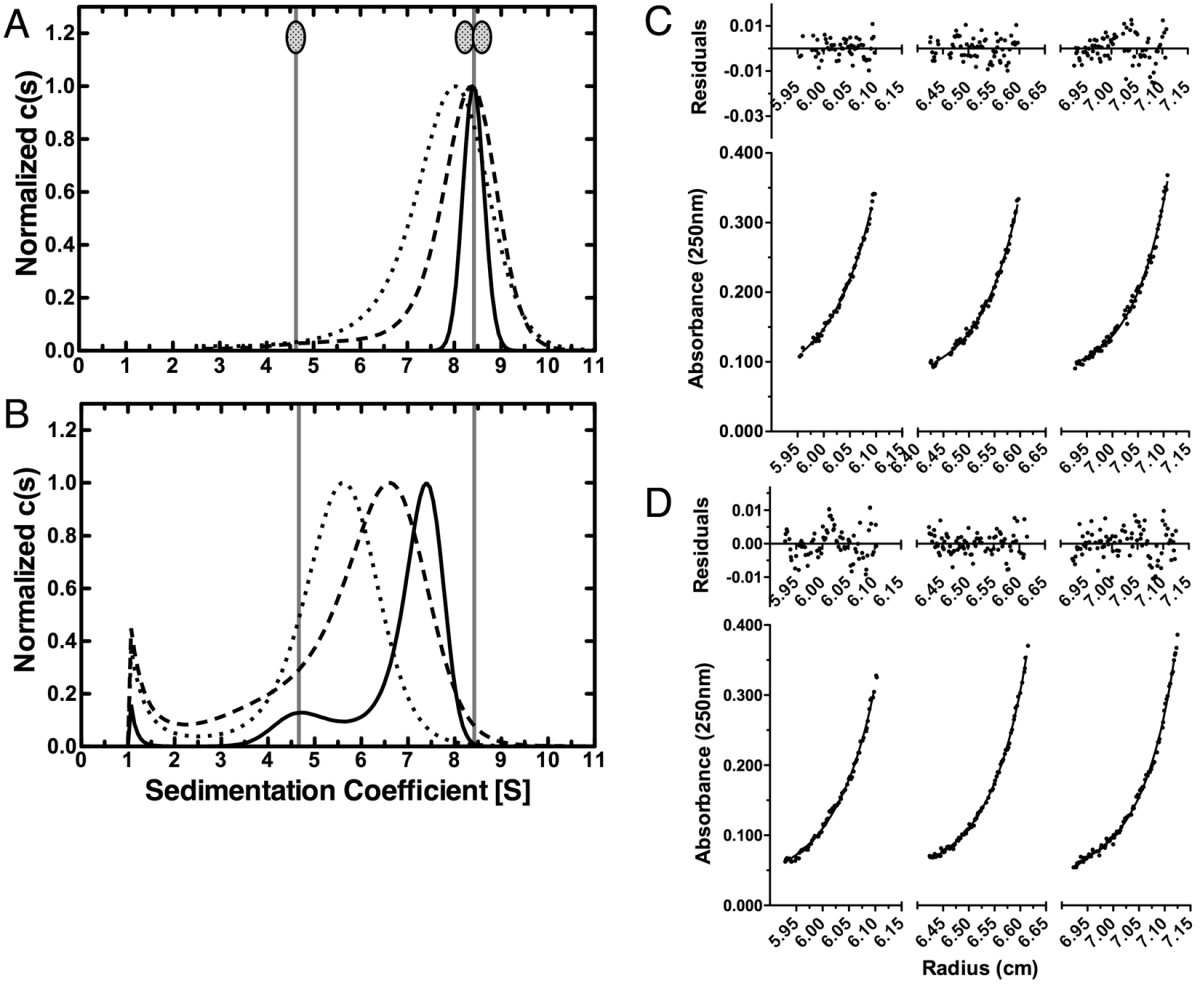
Sedimentation Analyses of SecA-N95 and SecA-N95ΔN Sedimentation velocity (Panels A and B) and equilibrium (Panels C and D) were used to evaluate the effect of N-terminal deletion on the dimerization of SecA-N95. For all experiments, the buffer used was 50 mM Tris-HCl, 100 mM KCl, 2 mM EDTA, 5 mM MgCl_2_, and pH 7.5. (**A**) Sedimentation velocity analysis of SecA-N95 at 31 µM (solid curve), 10.5 µM (dashed curve), and 2 µM (dotted curve). A sedimentation coefficient of 8.4 S is observed at concentrations of 10 µM and above and corresponds to the SecA-N95 dimer. (**B**) Sedimentation velocity analysis of SecA-N95ΔN at 26 µM (solid curve), 10 µM (dashed curve) and 2 µM (dotted curve). At 26 µM concentration, SecA-N95ΔN sediments with a major species at 7.4 S and a minor species at 4.6 S while at 2 µM it sediments with an overall coefficient of 5.6 S. In Panels A and B, the positions for sedimentation of the dimer at 8.4 S and monomer at 4.6 S are indicated. (**C**) Sedimentation equilibrium analysis of SecA-N95 was carried out in a 3-sector cell at rotor speeds of 7000, 10000, and 12000 rpm; the data from all nine curves were globally fit to a model of a single ideal species to yield a MW of 173.5 kDa (the theoretical dimer MW is 189.3 kD). Representative sedimentation curves for the equilibration at 10000 rpm are indicated; the other six curves are omitted for clarity. (**D**) Sedimentation equilibrium analysis of SecA-N95ΔN was carried out at rotor speeds of 10000, 12000, and 16000 rpm; the data from all nine curves were globally fit to a monomer-dimer equilibrium model, using a MW of 93.2 kDa, which yielded a dimer dissociation constant of 24.5 µM. Representative sedimentation curves for the equilibration at 12000 rpm are indicated; the other six curves are omitted for clarity. In Panels (C) and (D), the absorbance data are indicated by the circles and the fit to the data by the solid curves; the residuals are indicated above the data.

The analysis of SecA-N68 indicates that formation of the tetramer is completely dependent on intermolecular interactions with the unstructured N- and C-termini. The SecA-N68∆C construct exists as a mixture of monomer and dimers, indicating that the N-terminus on its own can only mediate dimer formation of SecA-N68. We tested the degree to which the 14-residue deletion at the N-terminus affected the formation of the SecA-N95 dimer. In a buffer consisting of 50 mM Tris-HCl, 100 mM KCl, 2 mM EDTA, 5 mM MgCl_2_, and pH 7.5, SecA-N95 sediments solely as a dimer at concentrations of 10 µM and above, whereas SecA-N95ΔN, under exactly the same conditions, sediments as a mixture of monomeric and dimeric species (Fig. 5A,B), indicating that SecA-N95 dimerization is weakened, but not abrogated, by the loss of the extreme N-terminus. To quantify the change in dimer dissociation constant, sedimentation equilibrium experiments were conducted with SecA-N95 at a concentration of 1.3 µM (2.5 mg/mL) and fit to a single ideal species with a molecular weight of 173.5 kDa (Fig. 5C); this indicates that the dimer dissociation constant for SecA-N95 is in the sub-micromolar range, consistent with previous studies^45,46^. At the same concentration, the SecA-N95ΔN construct did not yield a suitable fit as a single species, but yielded an excellent fit to a monomer-dimer equilibrium, with a dissociation constant of 24.5 µM (Fig. 5D). Therefore the N-terminus makes a strong contribution to dimerization of SecA-N95, but is not absolutely essential in the way it is for the formation of the SecA-N68 tetramer. Since the N-terminus alone is not completely responsible for mediating SecA-N95 dimer formation, the C-terminal domains of SecA must also contribute to the dimer interface.

### A novel structure for the SecA-N95 solution dimer

The link between the functional importance of the N-terminus and its role in SecA dimerization leads naturally to questions about the structure of the SecA solution dimer. A previous SAXS analysis on full-length SecA was completed before any crystal structures were available^24^. New SAXS data for both full-length SecA and SecA-N95 were collected to compare the two proteins and evaluate the fit of the crystallographic dimers to solution SAXS data for SecA-N95, since none of the crystallographic dimers contain the complete C-terminal linker and zinc-binding domain.

**Figure 6:**
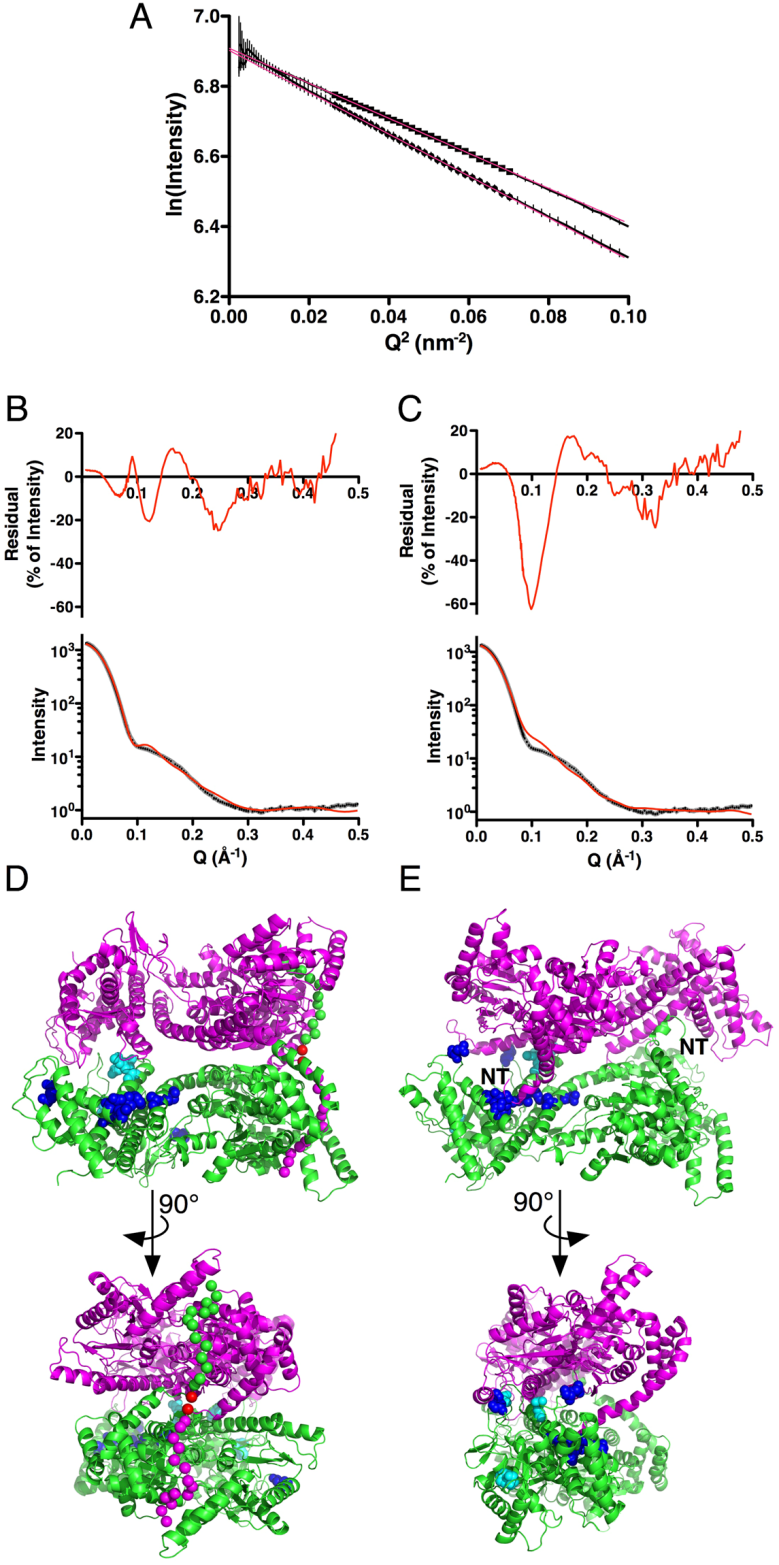
Parallel and Antiparallel Dimer Models (**A**) Guinier plot of the low angle SAXS data for full-length SecA (bottom curve) and SecA-N95 (top curve). For clarity, the curves have been normalized to the same *I*(0) value of 1000, and in both cases the standard deviation of the measurements are indicated by the vertical bars. Data points used for estimation of *R*_G_ are indicated by circles for full-length SecA and squares for SecA-N95; least-squares fits to these data are shown by the red lines. The analysis yielded an *R*_G_ of 42.3 ± 0.3 Å for full-length SecA and 38.6 ± 0.2 Å for SecA-N95. (**B**) Comparison of theoretical scattering from the parallel SAXS-derived dimer model of SecA-N95 with experimental SAXS data. The bottom panel shows the experimental data in grey, with standard deviations of the measurements as small black bars; the superimposed red curve is the theoretical scattering calculated using the FoXS server^60,66^. The top panel shows the residuals as a percentage of the intensity, on a linear scale. The *R*_G_ of the model is 38.1 Å and the fit yields a χ value of 10.8. (**C**) Comparison of theoretical scattering from the antiparallel *E. coli* dimer based on the crystallographic dimer present in the original *B. subtilis* SecA structure^28^ and modelled using the *E. coli* SecA-N68 crystal structure and homology models of the C-terminal domains. The *R*_G_ of the model is 39.8 Å and the fit yields a χ value of 17.6. (**D**) The parallel dimer with its long axis parallel to the page (top panel) and rotated 90° about a horizontal axis (bottom panel) to create a view down the two-fold rotation axis. The N-termini mediating dimer formation are indicated by magenta or green spheres at CA positions. The interactions between the N-termini and the opposite protomer are the same as those modelled for the interaction in the SecA-N68 tetramer, with adjustments of residues 15 through 18 to accommodate the somewhat different orientation of the protomers with respect to each other. Residue 15, the first residue present in the SecA-N68∆NC structure, is indicated with a red sphere. Sites of *in vivo* photo-activated cross-linking are indicated by residues highlighted with spheres and coloured cyan for one study^26^ and blue for a second study^25^. (**E**) The antiparallel dimer viewed down its two-fold rotation axis (top panel) and rotated 90° about a vertical axis; in this case the N-terminal amino acids of *E. coli* SecA from residue 3 (indicated by “NT”) onwards, were modelled from the *B. subtilis* structure. Cross-linking sites are indicated as in Panel D.

SAXS data were collected under conditions of protein concentration (approximately 6 mg/mL) and ionic strength (50 mM Hepes, 100 mM NaCl, pH 7.5) that favour the SecA dimer. The Guinier curves for SAXS data for full length SecA and SecA-N95 (Fig. 6A) have an extended linear region, indicating mono-disperse preparations: the curve for full-length SecA shows a small amount of curvature below a *Q* value of 0.14 nm^−1^ (Q^2^ of 0.02 nm^−2^), while SecA-N95 is linear past a *Q* value of 0.07 nm^−1^ (*Q* ^2^ of 0.005 nm^−2^). The high quality of the data allow for unambiguous calculation of the radius of gyration (*R*_G_) and detailed comparison to potential dimer models. For full-length SecA, an *R*_G_ of 42.3 ± 0.3 Å was similar to that observed previously^33^. For SecA-N95 the *R*_G_ was 38.6 ± 0.2 Å. SecA-N95 is truncated at residue 835 and is therefore missing 65 residues at the C-terminus, which includes a linker rich in hydrophilic residues followed by a 25 residue zinc binding domain^47^. The C-terminal linker and ZBD are most likely extended in solution.

The data for SecA-N95 were used to evaluate the fit of the available crystallographic dimers. In fact, none of the crystallographic dimers provides a completely convincing fit to the data (Table 2). The dimer that comes closest is the anti-parallel dimer from *B. subtilis* (PDB-ID 1M6N) which was observed in the original SecA structure^28^. To improve the fit, a model of similarly structured *E. coli* dimer was made using the domains from the SecA-N68 crystal structure combined with a homology model of the *E. coli* C-terminal domains using the *B. subtilis* structure as template (Fig. 6E). This decreased the χ value of the fit from 25.5 to 17.6, but there was still a systematic deviation around a *Q* value of 0.1 Å^-1^ (Fig. 6C), and the theoretical *R*_G_, at 39.8 Å, is larger than the experimentally observed *R*_G_ for *E. coli* SecA-N95, 38.6 Å. The SecA structure is flexible, particularly with respect to the PPXD, which is observed in a number of different states in the various crystal structures. On this basis, alternate symmetrical and asymmetrical conformations of the PPXD (i.e. dimer structures in which the PPXD was in the same or different conformations on each protomer), were tested in the context of the original *B. subtilis* dimer, but movement of the PPXD only worsened the fit to the SAXS data because changes from its original position in one or both of the protomers increased the *R*_G_ further away from the experimentally observed value. The fact that movement of the PPXD in the 1M6N dimer increases the *R*_G_ also means that a mixed population of these dimers (i.e. with the PPXD in various positions) will not improve the fit to the SAXS data.

**Table 2:** Evaluation of SecA Solution Dimers.

Organism	Structure	*R*_G_(Å)	^a^SAXSχ	N-term in Interface	^b^FRET χ^2^ value	Reference and Comments
*B. subtilis*	1M6N	38.1	25.5	Yes	1.1	Hunt *et al*.^28^
*E. coli*	1M6N (*E. coli* model)	39.8	17.6	Yes	1.1	SecA-N68 used to model *E. coli* protomer structure; 1M6N dimer interface
*B. subtilis*	2IBM	39.5	44.7	No	6.2	Zimmer *et al*.^68^
*B. subtilis*	3JV2	45.4	36.4	No	—	Zimmer and Rapoport, 2009
*M. tuberculosis* SecA1	1NKT AB dimer	46.3	50.5	No	6.7	Sharma *et al*.^63^
*M. tuberculosis* SecA1	1NKT AC dimer	39.8	31.9	(Yes)^c^	—	Sharma *et al*.^63^; similar arrangement to 1M6N dimer
*T. thermophilus*	2IPC	44.7	34.3	Yes	4.7	Vassylyev *et al*.^64^; parallel dimer
*E. coli*	2FSF	42.9	16.1	No	6.9	Papanikolau *et al*.^34^; PPXD from SecA-N68∆NC added to structure
*B. subtilis*	3JV2 SAXS-based Model	36.9	10.3	Yes	—	3JV2 protomer conformation (Zimmer and Rapoport)^48^; new dimer structure found using SAXS
*E. coli*	3JV2 SAXS-based Model	38.0	10.6	Yes	2.8	SecA-N68 and 3JV2 CT domains used to model *E. coli* protomer structure; SAXS-based dimer interface.

^a^The χ value as reported by the FoXS server^60,66^ after fitting the structures to SAXS data for *E. coli* SecA-N95.

^b^Fifteen FRET-based distance measurements were used from Auclair *et al*., as listed in Table 1 of the manuscript^22^. For each measurement, the χ^2^ value was calculated as the square of the difference between observed and theoretical distance, divided by the squared estimated FRET error. The value reported is the reduced χ^2^, namely the sum of the 15 values divided by 15.

^c^This dimer resembles the antiparallel 1M6N dimer but the N-terminus in the interface includes 17 N-terminal residues from the recombinant expression vector.

Based on the relatively poor fit of the crystallographic dimers to the SecA-N95 SAXS data, the available SecA-N95 protomer structures were used to look for alternative solution dimers using the SAXS data with GLOBSYMM^39^. Most of the dimers produced suffered from either severe steric clashes or had the N-termini pointing into the solvent, where they would be unable to participate in the dimer interface. However, using the protomer from the *B. subtilis* structure that contained the PPXD in an “open” conformation (PDB-ID 3JV2)^48^, a parallel dimer was found that provided a relatively good fit to the SAXS data (Table 2, Fig. 6B), combined with an absence of steric clashes. Furthermore, the N-terminus of each protomer was oriented in a manner that would allow interaction with the DEAD motor domain of the opposite protomer, similar to the interaction proposed for SecA-N68 oligomerization (Figs 3D and 6D).

The parallel SAXS-based dimer incorporates the same interface as the anti-parallel crystallographic dimer, and on this basis is also consistent with much of the previous work characterizing the structure of the solution dimer. This includes *in vivo* photo-activated cross-linking studies^25,26^: cross-linking sites identified in these studies are highlighted in the dimer structures in Fig. 6. Regarding the first study^26^, two of the sites, at positions 794 and 805, are in close proximity in the dimer interface of both the parallel and anti-parallel structures; the third site, at position 263, is present on the PPXD and for both dimers would require conformational changes in the PPXD for an interaction with the opposite protomer. In the second study^25^, a region containing a number of cross-linking sites is interacting with the N-terminus of the opposite protomer in the anti-parallel dimer; for the parallel dimer, this region is also in the interface and interacting with a loop comprising residues 792 to 801. Of the five potential crystallographic dimers, an extensive FRET study strongly supported the arrangement in the antiparallel *B. subtilis* dimer^22^. These distance measurements were used to compare the crystallographic dimers and SAXS-based model using a χ^2^ metric that includes the discrepancy between the observed and theoretical distances and the estimated distance error in the FRET measurement (Table 2). The original *B. subtilis* M6N dimer yields the best fit to these measurements (χ^2^ = 1.1), while the SAXS-based dimer comes second (χ^2^ = 2.8).

Overall, the SAXS-based parallel dimer offers an alternative to the *B. subtilis* anti-parallel arrangement that is in roughly similar broad agreement with the available data, although neither of the dimers provides full agreement with all of the data. The oligomerization of SecA-N68 demonstrates that there is a binding site for the N-terminus somewhere on either the PPXD or the DEAD motor domains. SAXS-based modelling shows that this same binding site could be used to mediate dimerization of SecA in a parallel arrangement.

## Discussion

Formation of the SecA-N68 tetramer is completely dependent on unstructured polypeptide at the N- and C-termini. This shows that the DEAD Motor domains, NBD1 and NBD2, plus the PPXD, have no tendency to self-associate, which is consistent with the crystallization of SecA-N68∆NC as a monomer. The SecA-N68 tetramer has a dimer-of-dimers symmetry; in addition, SecA-N68 constructs with unstructured regions at either the N- or C-terminus form dimers with different properties. Thus, biochemical data combined with the SAXS-based tetramer structure indicates two independent polypeptide binding sites on SecA-N68: one for the unstructured N-terminus, and a second site whose function and specificity is not clear. Currently there is no evidence that these binding sites actually function in translocation. However, given the important role of the N-terminus in SecA function, as well as the fact that SecA must translocate, and presumably bind to, unstructured preproteins, the ability of the SecA-N68 construct to mediate interactions with both the N-terminus and additional unstructured polypeptide has potential implications for the translocation mechanism that warrant further investigation.

SecA-N95 protomers have crystallized in a variety of arrangements, although none of them, with the possible exception of the anti-parallel dimer observed in the original *B. subtilis* crystal structure^28^ appear to be fully representative of the *E. coli* solution dimer. The antiparallel dimer is broadly consistent with biochemical studies of *E. coli* SecA, and provides a reasonable fit to the SAXS data for *E. coli*, although its *R*_G_ is somewhat larger than observed for the *E. coli* SecA-N95 construct. In the antiparallel arrangement, the N-terminus interacts with the C-terminal domains; this is obviously different from the N-terminal interaction that mediates oligomerization of SecA-N68, which lacks the C-terminal domains. Therefore, if the antiparallel dimer is representative of the solution dimer, there must be two binding sites for the N-terminus: one in the helical wing domains, and a second within SecA-N68.

SAXS data for SecA-N95, as well as the crystal structure of SecA-N68 and a conformation of the protomer observed in one of the *B. subtilis* structures, allowed us to model a SecA-N95 parallel dimer that facilitates an interaction between the N-terminus and the DEAD Motor. This parallel dimer has a *R*_G_ close to that experimentally determined, and makes an almost perfect match to the lower-angle SAXS data, which are characteristic of the overall shape of the scattering particle. The parallel dimer also provides an excellent match to the higher-angle SAXS data, which are more dependent on finer structural features of the particle, such as the exact conformation of the domains. In addition, this parallel dimer is consistent with much of the previous biochemical data, including *in vivo* cross-linking studies and FRET analysis^22,25,26^. Therefore, the parallel arrangement that is in relatively good agreement with the SAXS data offers an alternative dimer model that requires only a single binding site for the N-terminus of SecA, located somewhere within the domains of the SecA-N68 construct.

The proposed parallel arrangement is flexible. The SecA-N68∆NC construct shows no tendency to self-associate, so the flexible termini are completely responsible for the interactions. The SAXS-based models of the SecA-N68 tetramer and the SecA-N95 dimer show that it is possible for the globular subunits to adopt different relative orientations while maintaining the same interactions with the N-terminus, in this case modelled into the groove between NBD1 and NBD2 which was identified as a peptide binding “hotspot” using the CABS-Dock server^40,41^. With this type of interaction, the C-terminal domains could adopt different relative orientations, possibly explaining why the exact solution structure of the SecA dimer has been so difficult to define. In addition, this flexibility could enable dynamic interactions with SecYEG and preprotein.

The extreme N-terminus of SecA is critical for translocase function: removal of 14 N-terminal residues of SecA results in a protein that is unable to functionally complement the system. Other groups have made similar observations with N-terminal deletions or site-directed mutagenesis^14,29–32^. While there is evidence that the N-terminus has a critical role in membrane interactions^30–32^, this does not exclude other possible functions. For example, an interaction between the N-terminus and the DEAD Motor domain could serve to mediate SecA oligomerization during translocation, as well as regulate ATP binding or hydrolysis.

The second peptide binding site on SecA-N68, with which the C-terminal unstructured region of SecA-N68 interacts, is also of interest. The SAXS-based tetramer structure is consistent with this binding site being in the vicinity of the beta-strands connecting NBD1 to the PPXD. On this basis, the site could be the same as the peptide binding site observed in *B. subtilis* ^48^ or possibly the signal-sequence binding site in *E. coli* SecA, characterized by NMR^49^. The use of deletion constructs such as SecA-N68 will likely be helpful in detailed mapping and further characterization of these polypeptide binding sites.

## Materials and Methods

Chromatography resins were obtained from GE Healthcare. The Cibacron-blue affinity resin used for SecA purification was made by alkaline coupling of Cibacron Blue 3GA (Sigma) to Sepharose CL-6B. Approximately 145 g of hydrated Sepharose CL6B was mixed with 220 mL of 0.5 M NaCl and 8.4 g of Cibacron Blue dye; 9 mL of 10 M NaOH was added and the suspended resin was mixed on a shaker table for 60 minutes at 37 °C. The resulting blue gel was washed successively with 1 M NaOH, H_2_O, 60% ethanol, H_2_O, and finally 20% ethanol for storage.

### Cloning and Molecular Biology

Molecular cloning and mutagenesis was carried out using standard PCR-based protocols^50^ with PfuTurbo DNA polymerase (Stratagene). In all cases, results of the cloning and mutagenesis were confirmed by DNA sequencing.

### Expression and Purification of SecA Constructs

Wild-type SecA and SecA-N95 were expressed from plasmid pZ52^51^ in a BL21(DE3) background. SecA was precipitated from the crude cell extract using (NH_4_)_2_SO_4_ at 50% saturation and 4 °C. After centrifugation at 5000 x g, the (NH_4_)_2_SO_4_ pellet was re-dissolved in 50 mM Tris-HCl, pH 8.3, and supplemented with (NH_4_)_2_SO_4_ to bring the concentration to 1 M. The solution was applied to a 2.6 × 20 cm column of Fast Flow PhenylSepharose Hi-Sub resin, equilibrated with 50 mM Tris-HCl, 1 M (NH_4_)_2_SO_4_, pH 8.3, at 4 °C, and eluted with 10 mM Tris-HCl, pH 8.3. Fractions containing SecA were pooled and supplemented with 50 mM Tris-HCl, 1.5 M KCl, pH 8.5, to bring the final concentration of KCl to approximately 120 mM. This solution was applied to a 2.6 × 20 cm column of Cibacron Blue Sepharose CL6B, equilibrated with 50 mM Tris-HCl, 120 mM KCl, pH 8.5, and eluted with a 500 mL linear gradient ending in 50 mM Tris-HCl, 1.5 M KCl, pH 8.5. SecA-containing fractions were pooled and dialyzed against 50 mM Tris-HCl, pH 8.5, and subjected to anion exchange chromatography on MonoQ HP, in 50 mM Tris-HCl, pH 8.5, with a gradient of NaCl from 50 to 500 mM. The truncated fragment His_6_-SecA-N68 is comprised of residues 6 to 610 and includes a hexahistidine tag at the N-terminus with the sequence MHHHHHHLTK; this tag replaces the first 4 residues of SecA, which is MLIKLLTK. SecA-N68 was expressed and purified as previously described^33^.

Numerous SecA constructs were expressed as hexahistidine-tagged fusions incorporated into the pProEX-HTa vector (Invitrogen). In these cases, Ni-NTA affinity chromatography was used as the first purification step, which was followed by removal of the hexahistidine tag and linker by treatment with TEV protease. Removal of the affinity tag in this manner resulted in a leftover “GA” sequence at the N-terminus of the constructs. Depending on the construct, subsequent purification steps incorporated Cibacron Blue affinity, anion-exchange, and/or gel filtration chromatography.

### Analytical Gel Filtration Chromatography

Analytical gel filtration chromatography was carried out using a Superdex 200 HR 10/30 column. The running buffer was 50 mM Tris-HCl pH 7.5, 100 mM KCl, 1 mM EDTA, 5 mM MgCl_2_, and 5 mM β-mercaptoethanol, and the column was developed at a flow rate of 0.7 mL•min^−1^ at room temperature. Samples (50 µL) of the analyte or molecular weight standards were injected onto the column and absorbance was monitored at 280 nm. The molecular weight standards used were obtained from Sigma and included catalase (250 kDa), alcohol dehydrogenase (150 kDa), BSA (66 kDa), and carbonic anhydrase (29 kDa). Acetone (10 mg•mL-1) and Blue Dextran 2000 (GE Healthcare; 1 mg•mL-1) were used to determine the included and void volumes, respectively.

### Analytical Ultracentrifugation

Analytical ultracentrifugation experiments were conducted at 20 °C in a Beckman Optima XL-A analytical ultracentrifuge using an An-60 Ti four-place analytical rotor. Protein samples were extensively dialyzed against 50 mM Tris-HCl pH 7.5, 100 mM KCl, 2 mM EDTA, and 5 mM MgCl_2_. This buffer also included 5 mM TCEP-HCl to ensure the full reduction of free Cys residues. The final dialysis buffer was used as the reference solution.

For sedimentation velocity experiments, standard two channel (double-sector) epon-charcoal centerpieces with quartz windows were used. Protein solution (at various concentrations) and the reference solution were injected into the sample and reference cells. After thermal equilibration of the rotor at low speed (1000 rpm), samples were subjected to a high speed ranging from 25,000 rpm to 40,000 rpm depending on the expected size of the sedimenting protein species. Absorbance was monitored at 280 nm or 295 nm and higher for more concentrated protein samples so that initial absorbance readings were in the range from 0.15 to 0.6. Absorbance measurements were collected using a 0.002 cm radial step and averaged over three readings. Overall 30 scans were collected in intervals of 10 min. Data were processed and size distribution analyzed using SedFit^52^. The partial specific volume ($\bar{\nu }$) of each protein was calculated from the amino acid composition with SEDNTERP software (http://bitcwiki.sr.unh.edu/index.php/Main_Page). Using the same software, the solvent viscosity and density were calculated to be 1 mPa•s and 1.005 g•mL^−1^, respectively.

Sedimentation equilibrium experiments were conducted with six-channel cells consisting of epon-charcoal centerpieces with quartz windows and a path length of 1.2 cm. After reaching equilibrium at rotor speeds of 7,000, 10,000, 12,000, and 16,000 rpm, absorbance data at 250 nm were collected at 0.002 cm radial steps and averaged over ten readings. The absorbance data were analyzed using models built in Prizm 5 (Graphpad) with the following equations^53^.

A single ideal protein model was defined by equation 1.

1 $$C={C}_{0}\bullet \exp [\frac{{\omega }^{2}}{2RT}\bullet {M}_{obs}(1-\bar{v}\rho )\bullet ({x}^{2}-{x}_{0}^{2})]+{I}_{0}$$

In this expression, *C* is the concentration at radius *x*, *C*_0_ the concentration at reference radius × _0_, *ω* the angular velocity, $\bar{\nu }$ the partial specific volume of the analyzed protein, *M*_obs_ the molecular weight of the protein, *ρ* the solvent density, *T* the temperature in Kelvin, *R* the ideal gas constant, and *I*_0_ is the baseline offset. For self-associating proteins, a series of protomer:n-mer models (i.e. monomer-dimer, monomer-tetramer) with different values of n were also built; the association constants, *K*_A_, for these models are defined by Equation 2.

2 $${K}_{A}=\frac{{C}_{n-mer}}{{({C}_{monomer})}^{n}}$$

Equilibrium data of SecA proteins were then fit to the protomer:n-mer models using Equation 3.

3 $$\begin{array}{c}C=\{{C}_{0}\bullet \exp [\frac{{\omega }^{2}}{2RT}\bullet M(1-\bar{v}\rho )\bullet ({x}^{2}-{x}_{0}^{2})]\}\\ \quad +\{{C}_{0}^{n}\bullet {K}_{A}\bullet \exp [\frac{{\omega }^{2}}{2RT}\bullet nM(1-\bar{v}\rho )\bullet ({x}^{2}-{x}_{0}^{2})]\}+{I}_{0}\end{array}$$

In this equation, M is the molecular weight of the protomer and other terms are as defined above. The *K*_A_ values obtained by this equation have units of reciprocal absorbance, were then converted to the corresponding molar dissociation constant using an extinction coefficient for SecA-N95 of 26474 M^−1^•cm^−1^ at 250 nm.

### Crystal Structure Analysis of SecA-N68∆NC

To obtain high quality crystals, SecA-N68∆NC was modified with the following surface entropy-reducing (ER) mutations^37^: E55A, K56A, E58A, E196A, and E197A. SecA-N68∆NC was expressed in a BL21(DE3) background from vector pProEX-HTa (Invitrogen) as a hexahistidine-tagged fusion. After an initial Ni^2+^-affinity chromatography purification step, the protein was dialyzed against 50 mM Tris-HCl, 100 mM NaCl, 1 mM EDTA, 5 mM DTT, pH 8.0 and the affinity tag was removed with TEV protease. SecA-N68∆NC was further purified by anion exchange chromatography on a 1.6 × 10 cm column of Q-Separose HP, in a base buffer of 50 mM Tris-HCl, 1 mM EDTA, 2 mM DTT, pH 8.2; proteins were eluted with a linear NaCl gradient. Purified SecA-N68ΔNC-ER was concentrated to 15 mg•mL^−1^, dialyzed against 10 mM Tris-HCl, 2 mM DTT, pH 8.0, aliquoted and stored at −80 °C. SecA-N68ΔNC was crystallized against a reservoir of 0.1 M Tris-HCl pH 8.0, 18% PEG 3000 and 0.9% w/v cadaverine at 25 °C. Crystals were cryo-protected by passage through the reservoir solution supplemented with 20% PEG 200.

Data from crystals of SecA-N68∆NC were collected from beamline 08ID-1 of the Canadian Macromolecular Crystallography Facility at the Canadian Light Source. Data were processed with Mosflm^54^ and merged with Scala^55^. The structure was solved and refined using Phenix^56^ and COOT^57^. SecA-N68ΔNC was solved by molecular replacement using SecA-DM^58^ and the PPXD from *B. subtilis* ^28^.

### SAXS Data Collection and Analysis

The SAXS data for SecA-N68 were collected as previously described^33^. For full length SecA and SecA-N95, the purified proteins were concentrated to approximately 20 mg/mL and then, to remove any aggregated protein, were gel filtered using a Superdex SD200 10/30 column (GE Healthcare) in a running buffer of 50 mM Hepes, 100 mM NaCl, 5 mM NaN_3_, 25 mM β-mercaptoethanol, pH 7.4. The peak fractions were combined in each case to yield solutions with a protein concentration of 6.7 mg/mL for SecA and 5.8 mg/mL for SecA-N95 that were used for SAXS measurements. SAXS was recorded at BioCAT, Beam Line 18ID of the Advanced Photon Source (Argonne, Illinois USA) at a temperature of 20 °C. The buffer alone was measured first, followed by three measurements of the protein solution, and a final second measurement of the buffer alone. The data were reduced using Fit2D^59^ and additional processing carried out in Excel. Molecular weights of the SecA proteins were calculated based on their I(0) values compared to a maltose binding protein standard, measured under identical conditions.

### Modelling of the SecA-N68 Tetramer

For the SecA-N68 tetramer, the modelling process involved rigid-body fitting of the SecA-N68ΔNC crystal structure to the solution SAXS data, which yielded a number of different possible tetrameric solutions. These solutions were then assessed on the basis of whether the tetramer structures were consistent with subunit contacts that are mediated by the unstructured N- and C-termini.

The CABSdock server^40^ was used to locate the most likely sites for binding of the N- and C-terminal peptides which mediate formation of the tetramer. The CABSdock server^40^ only accepts receptor proteins with 500 residues or less, and therefore the search for peptide binding sites was done with two fragments from SecA-N68: the SecA-DM construct, consisting of NBD1 and NBD2, and a second construct consisting of NBD1 and the PPXD, as observed in the SecA-N68 crystal structure (the NBD2 domain was simply deleted from the SecA-N68 structure to create this construct). The sequences of the peptides used for the search were MLIKLLTKVFGSR (residues 1 to 13) for the N-terminus and EDALMRIFASDRVSGMMRK (residues 591 to 609) for the C-terminus. CABSdock works by running 10 docking trajectories each yielding 1000 receptor-peptide structures; out of these 10000 complexes, 1000 of the lowest energy complexes are selected. These solutions are then clustered based on their similarity in structure and position of the docked peptide; a metric termed the “cluster density” (equal to the number of structures in a given cluster, divided by the RMSD in the cluster) is used to rank the 10 different solutions. The detailed outputs from these analyses are presented in Supplemental Tables 1 to 4, and the positions of the bound peptides are illustrated in Supplemental Figs 1 to 4. In the case of the SecA-DM construct and the N-terminal peptide, 9 out of the 10 clusters contained the peptide in the cleft between NBD1 and NBD2; the other cluster had the peptide bound in the “clamp” region, roughly where the PPXD “stem” emerges from NBD1 (Supplemental Table 1 and Fig. 1). For the C-terminal peptide, 7 of the clusters had the peptide bound in the NBD1-NBD2 cleft, and the other 3 had the peptide located in the NBD1-PPXD “clamp” (Supplemental Table 2 and Fig. 2). Thus, the CABSdock server had identified two potential peptide binding regions in the SecA-DM construct. The results from the NBD1-PPXD construct, with both peptides (Supplemental Tables and Figs 3 and 4), indicated a peptide binding site on the PPXD (4 clusters out of 20); another peptide binding region in the NBD1-PPXD “clamp” (9 clusters out of 20); and 4 additional sites on the surface of NBD1. Overall, the analyses pointed towards the most likely regions for peptide binding residing in the NBD1-NBD2 cleft, the NBD1-PPXD “clamp” region, and the PPXD.

The SecA-N68 crystal structure was used as the protomer for rigid-body modelling against SAXS data using GLOBSYMM^39^. This process involves modelling of the tetramer using symmetry constraints: the protomer is rotated and translated to yield particles with the indicated (D2) symmetry; the quality of the models is evaluated based on their agreement with the SAXS data, along with evaluation of molecular contacts. Models that are structurally similar (an RMSD less than 9.5 Å) are grouped, and the best representative structure is output. For SecA-N68, the process yielded 11 models, listed in Supplemental Table 5, and illustrated in Supplemental Fig. 5. Since the oligomerization is mediated by the unstructured N- and C-termini, the models were inspected to find those that were structurally compatible with this constraint. Models 1 and 10 were not consistent with the biochemical data because both termini were pointing into the bulk solvent, and as such incapable of interacting with another protomer.

The remaining nine models contained termini that were positioned for interactions with other protomers, and each could potentially represent the solution tetramer, or at least a structure close to it. However, the CABSdock analysis indicated there were 3 likely binding sites for peptides, and to find tetramers that were compatible with the CABSdock analysis, the abilities of the N- and C-termini to interact with adjacent protomers at the CABSdock sites were evaluated. The PPXD is located on the outside of the tetramer in all the models, and only in Model 4 was there potential for the PPXD to mediate an interaction with the N-terminus of an adjacent protomer. For Model 4, the C-terminus was not well positioned for interaction with either of the two remaining binding sites. The other 8 models were evaluated based on whether the N-terminus could interact with the NBD1-NBD2 cleft, and the C-terminus with the NBD1-PPXD clamp region. In all the models except for Model 2 and Model 6, one or both of these interactions was not possible (Supplemental Table 5). However, Models 2 and 6, in which the protomers are arranged in a similar manner, satisfy the SAXS data and the protomers are arranged in such a way that the N- and C-termini can simultaneously interact with the most likely peptide binding sites identified by CABSdock; Model 6 also had the best agreement with the SAXS data, and no atomic overlaps.

### Evaluation and Modelling of SecA-N95 Dimers

For the SecA-N95 solution dimer, a number of potential models were available from SecA crystal structures, as listed in Table 2. Using the FoXS server^60^, the models were evaluated based on their radius of gyration (*R*_G_) and their overall agreement to the SAXS data for SecA-N95. The χ value for the agreement between a model and SAXS data is defined as:

4 $$\chi =\sqrt{\frac{1}{S}{\sum _{i=1}^{S}(\frac{{I}_{\exp }({q}_{i})-cI({q}_{i},{c}_{1},{c}_{2})}{\sigma ({q}_{i})})}^{2}}$$

where *S* is the number of data points, *I*_exp_(*q*_i_) and *I*(*q*_i_, *c*_1_, *c*_2_) are the experimental and calculated scattering values, *σ*(*q*_i_) is the estimated error, and *c* is a scale factor; *c*_1_ and *c*_2_ are corrections for excluded volume and hydration layer density. When required, homology modelling was carried out using Modeller^61^.

The experimental *R*_G_ for SecA-N95 was 38.6 ± 0.2 Å. Of the crystallographic dimer models listed, only the 1M6N, 2IBM, and 1NKT “AC” (where “AC” refers to the protein chains in the crystal forming the dimer) structures have *R*_G_ values that are close to the experimental, and on this basis are the only candidates for the solution dimer. The 2IBM structure has a relatively high χ value (44.7) indicating a poor overall fit to the SAXS data, and the N-termini are not positioned to mediate protomer interactions; therefore, the 2IBM structure is an unlikely model for the solution dimer. The 1M6N (*B. subtilis*) and 1NKT AC (*M tuberculosis*) structures have similar anti-parallel protomer arrangements, with the N-terminus of SecA interacting with C-terminal residues; however the 1NKT N-terminus includes 17 additional residues from the expression vector. In summary, based on the SAXS data and the requirement for the N-terminus to participate in the dimer interface, only the 1M6N dimer appears to be a viable model for the solution dimer. The overall fit to the SAXS data (χ = 25.5) was improved (χ = 17.6) by homology modelling the *E. coli* structure using the *B. subtilis* 1M6N structure as a template.

To look for other potential dimer structures, the various SecA protomer structures were used for rigid-body modelling to the SAXS data with GLOBSYMM^39^. Some of the structures resembled the 1M6N dimer, but most of the structures with suitable matches to the data had bad atomic overlaps or the N-termini were not positioned to contribute to protomer-protomer interactions. However, with the protomer from the *B. subtilis* 3JV2 structure^48^, a novel parallel dimer structure with an *R*_G_ of was 36.9 Å and χ of 10.3 was obtained; the arrangement of the protomers in this structure was such that the N-termini could contribute to subunit interactions by binding to the NBD1-NBD2 cleft region.

The crystallographic dimer models had been previously evaluated in a comprehensive FRET study^22^ and only the *B. subtilis* model appeared to exhibit a suitable agreement with the FRET data, as assessed in the original manuscript. To include the novel SAXS-based dimer in these results, we used the measured FRET distances from the study by Auclair *et al*.^22^, and estimated errors to calculate a reduced χ^2^ value, reported in Table 2 and defined by Equation 5:

5 $${\chi }^{2}=\frac{1}{N}\sum _{i=1}^{N}\frac{{(FRE{T}_{i}-DIS{T}_{i})}^{2}}{{\sigma }_{i}^{2}}$$

where N is the number of measurements, *FRET*_*i*_ is the distance determined by FRET, *DIST*_*i*_ is the distance between CA atoms of the labelled residues, and σ_i_ is the estimated error in the FRET measurement.

### *In Vivo* SecA Complementation Assay

The plasmids used for the complementation assays were pZ52 (which expresses full-length SecA, and was originally called pT7SecA2^62^) and derivatives of pZ52: pJZ7-N95 (expresses SecA-N95), and pAK-SecA-N95ΔN14 (expressed SecA-N95ΔN), and pAK-SecA∆N14 (expresses SecA∆N). Plasmid pJZ7-SecA-N95 is a derivative of pZ52 (originally pT7SecA2) that carries a stop codon after residue 835. Plasmids pAK-SecA∆N14 and pAK-SecA-N95ΔN14 were constructed from pZ52 and pJZ7-N95 by removal of the nucleotide sequence corresponding to the first 14 N-terminal residues of SecA using the primers F-AK-N95ΔN14 (5′-TGAGATTTTATTATGGATCGCACCCTGCGCCGGATG) and R-AK-N95ΔN14 (5′-CATAATAAAATCTCAAACGCCCCGCGTTGC). For *in vivo* analysis, the plasmids were transformed into *E. coli* BL21.19(DE3) strain [secA13(Am) supF(Ts) trp(Am) zch::Tn10 recA::CAT clpA::KAN]^43^. The constructs were grown in LB media containing ampicillin (100 µg•mL^−1^) and kanamycin (40 µg•mL^−1^) for 12 hours at 27 °C while shaking. After growth, the cell cultures were normalized to an OD_600_ of 1 by addition of LB media. The cell cultures were subsequently serially diluted in 10-fold steps with LB media to make 6 concentrations. 5 μL of each dilution was spotted on LB agar replica plates containing 100 µg•mL^−1^ ampicillin and 40 µg•mL^−1^ kanamycin. The plates were incubated at 28 °C or 42 °C for 30 hrs.

### Data Availability Statement

The coordinates and structure factors for the SecA-N68∆NC construct have been deposited in the Protein Data Bank with ID 5K9T. Molecular models for the SecA-N68 tetramer and SecA-N95 dimer, as well as the SAXS data used for modelling, are available from the corresponding author on request.

## Electronic supplementary material

Supplementary Information
