# Supplementary material for: An alternate mode of oligomerization for E. coli SecA
Source: Sci Rep. 2017 Sep 18;7:11747. doi: 10.1038/s41598-017-11648-5 (PMC5603524; doi:10.1038/s41598-017-11648-5)
Supplement: Supplementary file 1 — Supplementary Information [file 41598_2017_11648_MOESM1_ESM.pdf]

An alternate mode of oligomerization for *E. coli* SecA

Aliakbar Khalili Yazdi, Grant C. Vezina, and Brian H. Shilton*

Dept. of Biochemistry, University of Western Ontario, London Ontario N6B 2G3 Canada

Supplementary Material

Supplementary Table 1: Docking of SecA N-terminus to NBD1-NBD2

Cluster	Number[b]	RMSD[c]	Density[d]	Contact Residues[e]
1	138	3.2	43.6	NBD1: 105 to 145; NBD2: 485 to 525
2	157	4.4	35.9	NBD1: 80 to 145; NBD2: 495 to 580
3	164	5.6	29.2	NBD1: 75 to 145; NBD2: 495 to 580
4	205	8.4	24.5	NBD1: 135 to 225; NBD2: 455 to 515
5	56	2.4	22.9	NBD1: 75 to 145; NBD2: 495 to 585
6	73	3.6	20.4	NBD1: 75 to 145; NBD2: 510 to 585
7	74	5.9	12.6	NBD1: 75 to 145; NBD2: 510 to 585
8	46	4.2	10.9	NBD1: 75 to 145; NBD2: 495 to 585
9	43	5.7	7.6	NBD1: 110 to 145; NBD2: 485 to 585
10	44	7.1	6.2	NBD1: 75 to 145; NBD2: 485 to 585

[a]The receptor for docking consisted of SecA residues 15 to 227 and 368 to 590 containing NBD1 and NBD2, but not the PPXD.
[b]The number falling in a particular cluster from the 1000 lowest energy complexes.
[c]The average RMSD for the cluster.
[d]Complexes are ranked based on the cluster density, which is equal to the number in the cluster divided by the RMSD in the cluster.
[e]Contact residues fall in the indicated ranges.

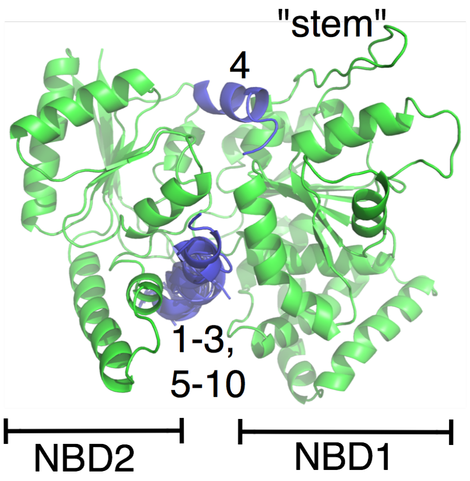

Supplementary Figure 1: Docking of SecA N-terminus to NBD1-NBD2
The locations of the docked N-terminal peptide (sequence MLIKLLTKVFGSR) are shown for each of the representatives of the 10 clusters listed in Table 1. The “stem” is the β-hairpin that would normally carry the PPXD.

Supplementary Table 2: Docking of SecA-N68 C-terminus to NBD1-NBD2[a]

Cluster	Number[b]	RMSD[c]	Density[d]	Contact Regions[e]
1	106	3.0	35.9	NBD1: 75 to 145; NBD2: 485 to 580
2	102	3.1	33.3	NBD1: 130 to 225; NBD2: 460 to 510
3	126	5.0	25.4	NBD1: 75 to 145; NBD2: 485 to 580
4	145	6.0	24.3	NBD1: 125 to 230; NBD2: 460 to 570
5	95	4.4	21.7	NBD1: 75 to 145; NBD2: 490 to 580
6	135	7.3	18.6	NBD1: 75 to 145; NBD2: 490 to 580
7	117	9.6	12.2	NBD1: 130 to 260; NBD2: 480 to 570
8	62	5.2	12.0	NBD1: 75 to 145; NBD2: 490 to 580
9	60	6.2	9.7	NBD1: 75 to 145; NBD2: 485 to 580
10	52	6.3	8.2	NBD1: 75 to 145; NBD2: 485 to 580

[a]The receptor for docking consisted of SecA residues 15 to 227 and 368 to 590 containing NBD1 and NBD2, but not the PPXD.
[b]The number falling in a particular cluster from the 1000 lowest energy complexes.
[c]The average RMSD for the cluster.
[d]Complexes are ranked based on the cluster density, which is equal to the number in the cluster divided by the RMSD in the cluster.
[e]Contact residues fall in the indicated ranges.

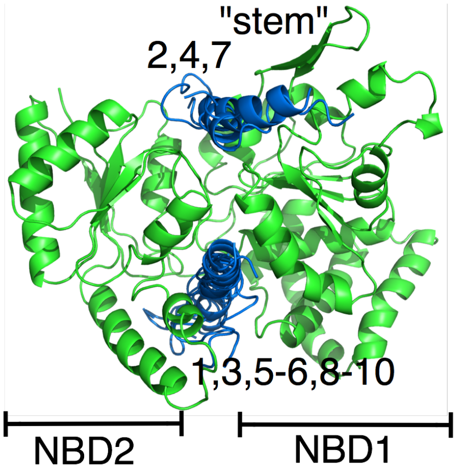

Supplementary Figure 2: Docking of SecA-N68 C-terminus to NBD1-NBD2
The locations of the docked C-terminal peptide (sequence EDALMRIFASDRVSGMMRK) are shown for each of the representatives of the 10 clusters listed in Table 2.

Supplementary Table 3: Docking of SecA N-terminus to NBD1-PPXD[a]

Cluster	Number[b]	RMSD[c]	Density[d]	Contact Regions[e]
1	103	1.87	55.0	PPXD: 260 to 355
2	80	2.92	27.4	PPXD: 270 to 315
3	129	5.76	22.4	PPXD: 270 to 355
4	74	4.19	17.7	NBD1: 155-230; PPXD: 360 to 370
5	83	8.92	9.3	NBD1: 75 to 145 , 205 to 215, 395 to 420
6	54	6.47	8.3	NBD1: 105 to 220
7	59	7.54	7.8	NBD1: 135 to 230, 345 to 370
8	107	13.86	7.7	NBD1: 225 to 245 and PPXD: 335 to 370
9	74	15.30	4.8	NBD1: 135 to 225
10	25	14.70	1.7	NBD1: 20 to 100

[a]The receptor for docking consisted of SecA residues 15 to 420 containing NBD1 and the PPXD.
[b]The number falling in a particular cluster from the 1000 lowest energy complexes.
[c]The average RMSD for the cluster.
[d]Complexes are ranked based on the cluster density, which is equal to the number in the cluster divided by the RMSD in the cluster.
[e]Contact residues fall in the indicated ranges.

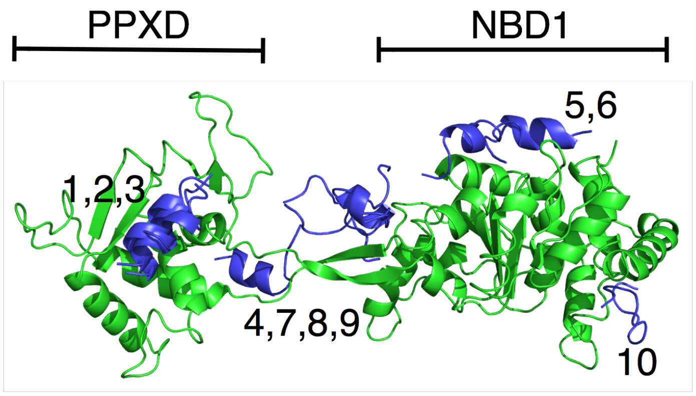

Supplementary Figure 3: Docking of SecA N-terminus to NBD1-PPXD
The locations of the docked N-terminal peptide (sequence MLIKLLTKVFGSR) are shown for each of the representatives of the 10 clusters listed in Table 3.

Supplementary Table 4: Docking of SecA-N68 C-terminus to NBD1-PPXD[a]

Cluster	Number[b]	RMSD[c]	Density[d]	Contact Regions[e]
1	118	5.1	23.2	NBD1: 20 to 125, 386 to 387
2	115	6.5	17.6	NBD1: 80 to 145, 210 to 215, 390 to 415
3	85	7.4	11.6	NBD1: 130 to 230, 330 to 370
4	37	3.8	9.8	NBD1: 130 to 230, 330 to 350
5	100	13.4	7.5	PPXD: 230 to 355
6	75	10.4	7.2	NBD1: 160 to 230, 330 to 370
7	70	17.4	4.0	NBD1: 20 to 125, 386 to 387
8	42	11.0	3.8	NBD1: 185 to 225, 370 to 385
9	35	9.1	3.8	NBD1: 130 to 230, PPXD 330 to 375
10	23	6.4	3.6	NBD1: 130 to 230, PPXD 330 to 380

[a]The receptor for docking consisted of SecA residues 15 to 417 containing NBD1 and the PPXD.
[b]The number falling in a particular cluster from the 1000 lowest energy complexes.
[c]The average RMSD for the cluster.
[d]Complexes are ranked based on the cluster density, which is equal to the number in the cluster divided by the RMSD in the cluster.
[e]Contact residues fall in the indicated ranges.

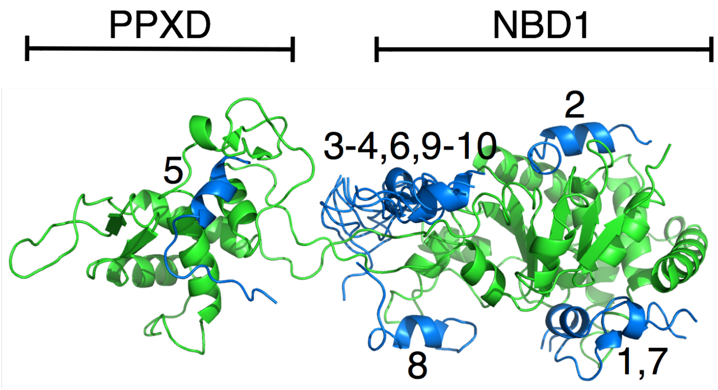

Supplementary Figure 4: Docking of SecA-N68 C-terminus to NBD1-PPXD
The locations of the docked C-terminal peptide (sequence EDALMRIFASDRVSGMMRK) are shown for each of the representatives of the 10 clusters listed in Table 4.

Supplementary Table 5: SecA-N68 Tetramers Modelled by Rigid-Body Fitting to SAXS Data

Model[a]	χ[b]	Overlap Penalty[c]	Protomer-Protomer Distance (Å)[d]	
			N-terminus to NBD1-NBD2 cleft	C-terminus to NBD1-PPXD clamp region
1	2.4	0.000	>63, C	>68, C
2	2.1	0.024	17, D	34, C
3	1.7	0.000	37, D	>35, C
4	2.1	0.001	>35, D	37, C
5	1.9	0.134	>32, D	36, C
6	1.5	0.000	28, C	31, B
7	2.2	0.551	>38, C	28, D
8	2.2	0.592	>37, D	29, B
9	1.6	1.052	>46, B	34, C
10	1.9	0.775	>64, D	>65, D
11	2.5	0.003	>54, C	>46, C

[a]Each model represents a group of solutions with an RMSD below 9.5 Å.
[b]The χ value reports on the agreement between the model and the SAXS data, and was calculated using Crysol (Svergun, Barberato, and Koch 1995). The formula for χ is provided in the Methods.
[c]The overlap penalty was calculated by GLOBSYMM, and represents steric clashes in the calculated structure.
[d]The distances are direct from the A-Chain N-terminal or C-terminal residue (15 or 590) CA atom, to the closest neighbouring Val514 (representing the NBD1-NBD2 cleft region) or Ile225 (representing the clamp region in the vicinity of the stem) CA atom; the identity of the chain containing the closest Val514 or Ile225 is indicated along with the distance. Where the direct distance passes through protein, the surface path travelled by the polypeptide would have to be much longer, and this is indicated by the "greater than" (>) sign.

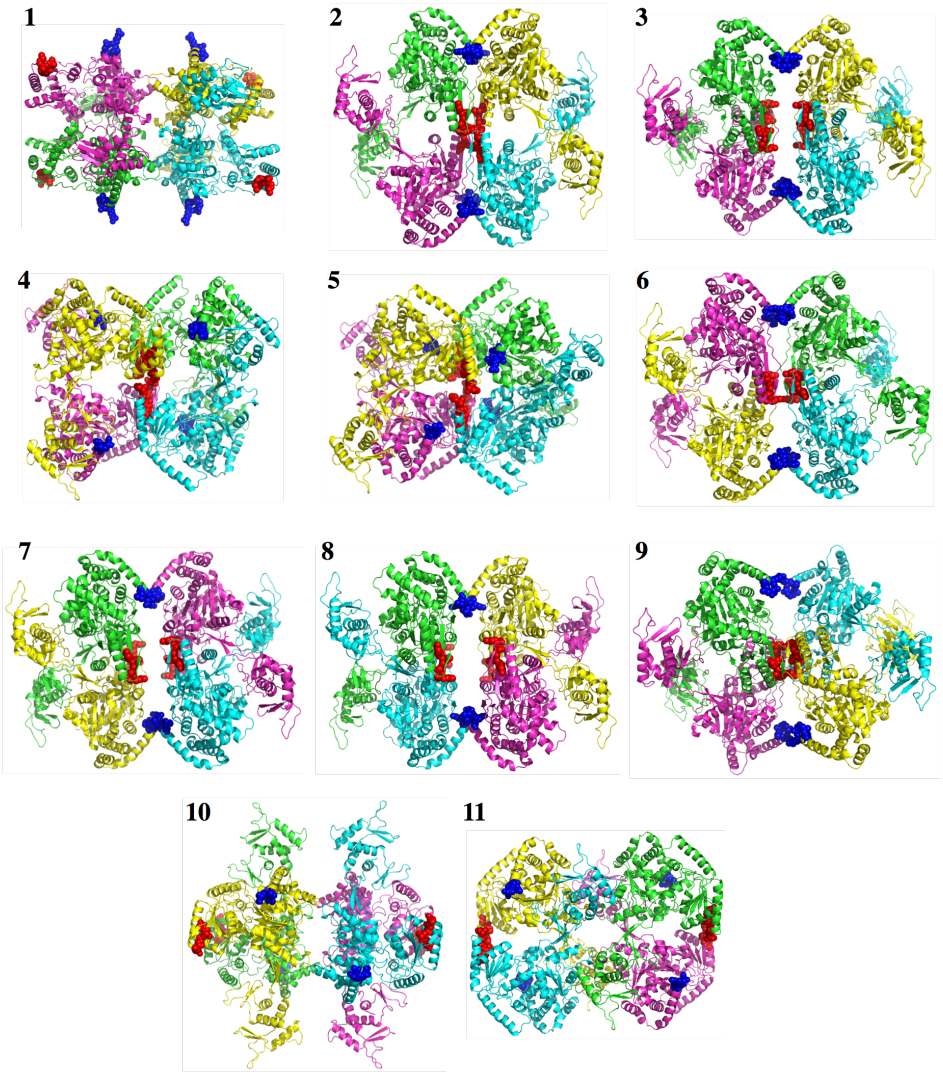

Supplementary Figure 5: SecA-N68 Tetramer Structures from Rigid Body Modeling
The program GLOBSYMM (Petoukhov and Svergun, 2005) was used for rigid body modeling of the SecA-N68 tetramer starting from the SecA-N68 crystal structure. The eleven models output by GLOBSYMM are illustrated, each one representing a cluster of similar solutions with RMSD less than 9.5 Å; the properties of the individual models are listed in Supplemental Table 5. All models have similar dimensions and overall shape, and all have the PPXD domains positioned on the exterior of the particle. Two models, represented by #2 and #6, have the SecA-N68 protomers positioned such that the N- and C-termini are able to interact with the NBD1-NBD2 cleft region, and the NBD1-PPXD clamp region, respectively.
